# Commentary: Correlation analysis of serum vitamin D levels and post-operative cognitive disorder in elderly patients with gastrointestinal tumor

**DOI:** 10.3389/fpsyt.2022.971412

**Published:** 2022-10-28

**Authors:** Nikhil Ravindranath Tondehal, Saadiya Hawa, Anem Sajid Malik, Kazi Nadia Hamid, Ashley Malekunnel, Mahwish Adnan, Chintan Trivedi, Zeeshan Mansuri, Shailesh Jain

**Affiliations:** ^1^Department of Psychiatry, Mount Sinai Beth Israel, New York, NY, United States; ^2^Department of Epidemiology and Population Health, Stanford University, Palo Alto, CA, United States; ^3^King Edward Medical University, Lahore, Pakistan; ^4^Sylhet MAG Osmani Medical College, Sylhet, Bangladesh; ^5^Amala Institute of Medical Sciences, Thrissur, Kerala, India; ^6^Department of Psychiatry, Texas Tech University of Health Sciences, Midland, TX, United States; ^7^Department of Psychiatry, Boston Children's Hospital, Boston, MA, United States

**Keywords:** POCD, gastrointestinal surgery, abdominal surgery, vitamin D level deficiency, cognitive change

Dear Editor,

We read with great interest the article, ‘Correlation Analysis of Serum Vitamin D Levels and Post-operative Cognitive Disorder (POCD) in Elderly Patients With Gastrointestinal Tumor' (1). This relevant article has explored the ongoing discussion regarding Vitamin D's multiple roles in maintaining health.

We have the following additional thoughts. The study missed addressing the complications faced during recovery from the surgery. Examples that can influence cognition are anesthesia recovery and electrolyte imbalance because of fluid loss during or after the surgery. Also, body weight plays a role in anesthesia recovery, i.e., lipid-soluble anesthetics with redistribution may affect a smooth recovery and result in continued confusion (2). The study failed to consider the association between the different anesthesia depths and POCD (3). The study misses considering the role of post-operative pain management in altering cognition (4). Elderly patients with gastrointestinal tumors may have fat depletion, influencing the absorption of fat-soluble vitamins such as Vitamins A, D, E, and K (5). Nutrient absorption is affected in most gastrointestinal tumors, especially fat absorption (6). Vitamins D, A, and K have antioxidant properties that influence post-surgery recovery (7, 8). Therefore, one way to identify absorption abnormalities could be to check the levels of other fat-soluble vitamins (A, E, and K). These findings suggest that low Vitamin D levels could be an expected and coincidental finding (9).

As Major Depressive Disorder affects cognition, screening patients for pre-existing depression could have been informative (10). The study discusses different confounders and mentions age and sex as significant confounders. However, the article does not clarify whether the odds ratios presented are crude or adjusted using multivariate logistic regression. In addition, women are more prone to osteoporosis and low vitamin D levels after menopause (11). It would be helpful to know the extent of confounding by reviewing the crude and adjusted odds ratios. Controlling for factors mentioned above (depression, anesthesia recovery, and pain management) would help provide a robust result that would assist the clinicians.

We believe that addressing the above issues will further improve the impact of this study.

## Author contributions

NT, SH, and ASM wrote the initial manuscript. AM and KH searched relevant literature and added references. NT, MA, CT, ZM, and SJ further proofread and edited the manuscript. All authors contributed to the article and approved the submitted version.

## Conflict of interest

The authors declare that the research was conducted in the absence of any commercial or financial relationships that could be construed as a potential conflict of interest.

## Publisher's note

All claims expressed in this article are solely those of the authors and do not necessarily represent those of their affiliated organizations, or those of the publisher, the editors and the reviewers. Any product that may be evaluated in this article, or claim that may be made by its manufacturer, is not guaranteed or endorsed by the publisher.
